# A Simple 3-Parameter Model for Cancer Incidences

**DOI:** 10.1038/s41598-018-21734-x

**Published:** 2018-02-21

**Authors:** Xiaoxiao Zhang, Holger Fröhlich, Dima Grigoriev, Sergey Vakulenko, Jörg Zimmermann, Andreas Günter Weber

**Affiliations:** 1 Bonn-Aachen International Center for Information Technology, Dahlmannstraße 2, Bonn, 53113 Germany; 2 Department of Medicine II, Klinikum Rechts der Isar, Technische Universität München, München, 81675 Germany; 3 German Cancer Consortium (DKTK), German Cancer Research Center (DKFZ), Heidelberg, 69120 Germany; 4 UCB Biosciences GmbH, Alfred-Nobel-Straße 10, Monheim, 40789 Germany; 5 CNRS, Mathématiques, Université de Lille, Villeneuve d’Ascq, 59655 France; 6 Institute for Mechanical Engineering Problems, Russian Academy of Sciences, Saint Petersburg, Russia; 7 Saint Petersburg National Research University of Information Technologies, Mechanics and Optics, Saint Petersburg, Russia; 8 Institut für Informatik II, Universität Bonn, Friedrich-Ebert-Allee 144, Bonn, Germany

## Abstract

We propose a simple 3-parameter model that provides very good fits for incidence curves of 18 common solid cancers even when variations due to different locations, races, or periods are taken into account. From a data perspective, we use model selection (Akaike information criterion) to show that this model, which is based on the Weibull distribution, outperforms other simple models like the Gamma distribution. From a modeling perspective, the Weibull distribution can be justified as modeling the accumulation of driver events, which establishes a link to stem cell division based cancer development models and a connection to a recursion formula for intrinsic cancer risk published by Wu *et al*. For the recursion formula a closed form solution is given, which will help to simplify future analyses. Additionally, we perform a sensitivity analysis for the parameters, showing that two of the three parameters can vary over several orders of magnitude. However, the shape parameter of the Weibull distribution, which corresponds to the number of driver mutations required for cancer onset, can be robustly estimated from epidemiological data.

## Introduction

Cancers arise after accumulating epigenetic and genetic aberrations^1–3^. Earlier studies established a power law model on the basis of multi-stage somatic mutation theory to explain age-dependent incidences^4–6^ for several cancer types. As noted by Hornsby *et al*.^7^ in the context of classical epidemiological studies most cancers occur with the same characteristic pattern of incidence, and the simplicity of this pattern is in contrast to the perceived complexity of carcinogenesis. Orthogonal to these age stratification of different cancer types, Tomasetti and Vogelstein^8^ (with follow-ups^9,10^) reported a significant association between life time caner risk and stem cell divisions and concluded the latter substantially contributes to the former. Challenging the conclusion of Tomasetti and Vogelstein^8^ of a high-intrinsic cancer risk Wu *et al*.^11^ subdivided cancer risk into extrinsic and intrinsic risk, arguing extrinsic factors contribute more to cancer risks than intrinsic factors do. Based on a mechanistic model of accumulated mutations, these authors provided a recursion formula for theoretical life time intrinsic risk (tLIR) parameterized by age *a*. This recursion formula has the closed form solution ${\rm{tLIR}}(a)=1-{\mathrm{(1}-{\mathrm{(1}-{\mathrm{(1}-r)}^{{\mathrm{log}}_{2}S+d\cdot a})}^{k})}^{S}$, where *S* can be interpreted as the numbers of stem cell, *d* as the stem cell division rate, *k* as number of driver events required for cancer onset and *r* as the mutation rate per division. They reported that tLIR goes outside of the plausible range of empirical cancer risks by studying several pairs of values for two parameters (mutation rate and driver gene mutations) concluding that there is a substantial contribution of extrinsic risk factors to cancer development. However, this conclusion only holds in the studied parameter space and when parameters for all cancer types are treated uniformly. By performing a systematic grid search in the space of biologically plausible parameter values we showed that tLIR can be close to empirical risk for different cancer types (*R*^2^ > 0.85). If the extrinsic risk factor is computed by simply setting it to a complement of 1 for the intrinsic risk factor as performed by Wu *et al*. it will be concluded that there is a *possibility* of high intrinsic risk, so that one of the presented arguments by Wu *et al*.^11^ is fallacious.

On a pure mathematical side, we show that a scaled Weibull function with 3 parameters approximates the 4-parameter mechanistic tLIR model. On an epidemiological data analytical side, this simple 3-parameter model excellently agrees with age-dependent cancer incidence curves among 18 common solid cancers even when variations due to different locations, races, or periods are taken into account. With this model, we study the relationship between cancer risk and stem cell divisions, the high correlation between the two entities reported by previous studies^8,10^ breaks down when considering age stratified data.

## Results

### Approximation of tLIR model by a scaled Weibull function

As is derived in the Materials and Methods the 4-parameter mechanistic tLIR model can be approximated by a scaled Weibull function with 3 parameters:

1 $${\rm{tLIR}}(a)\approx P\cdot {\rm{Weibull}}(\lambda ,\,k)(a),$$

assuming that *λ* is defined by

2 $$S{(rd)}^{k}={\lambda }^{-k}P\mathrm{.}$$

Here ${\rm{Weibull}}(\lambda ,\,k)(a)=1-{e}^{-{(a/\lambda )}^{k}}$ is the cumulative distribution function of the Weibull distribution, and *P* is the number of independent parallel processes, which e.g. can be interpreted as cell population at risk^12^. Whether the total tissue cells or only a fraction of stem cells are susceptible for cancer risk is unclear^13,14^. If one sets *P* = *S* then *rd* = *λ*^−1^. However, other possible choices for *P* allow to account for other factors such as the selection of mutations^15,16^, the stem cell microenvironment^17,18^, and tissue architecture^19–22^, or effects of clonal expansion^23–25^. Models incorporating clonal expansion have additional parameters such as the number of clonal copies. Reducing the dimensions of such complexed models results in tLIR, in which *S* is interpreted as number of independent clusters after clonal expansion rather than the number of stem cells, *r* and *d* denote “net” mutation and division rate of independent clusters at average level rather than those of single cells. Whereas a precise analysis of models for clonal expansion will be the topic of future work, these considerations show that when using the scaled Weibull distribution, prior knowledge on the parameter ranges is not necessary. This is indeed one benefit of scaled Weibull function comparing to tLIR model which requires a biologically reasonable guessing on stem cell numbers, mutation rate, cell division rate and number of driver mutations. The Weibull distribution is a special case of the generalized extreme value distribution (GEV)^26^. The GEV distribution plays the same role within extreme value statistics as the normal distribution does in average value statistics. It results in the limit distribution being maximized over many independent and identically distributed random variables, thus becoming the default model for the accumulation of micro events which finally leads to a macro event. The GEV is the limit distribution when one takes the maximum (and not the sum) of many independent and identically distributed random variables, thus being the default model for the accumulation of micro events which finally lead to a macro event. Accordingly, the Weibull distribution is not just a distribution providing a good empirical fit, but can be seen as justifiable for use in a plausible causative model of cancer genesis.

### Fitting empirical incidence rates with scaled Weibull function

**Figure 1:**
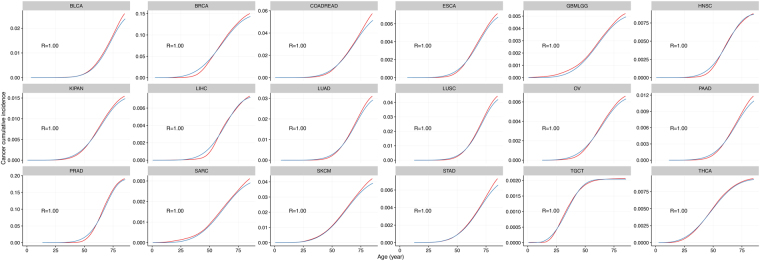
Empirical cumulative cancer incidence data are consistent with the Weibull cumulative probability function in 18 cancers (data for ages up to 85 years old). Empirical (blue line) and Weibull function-fitted (red line) cancer cumulative incidence curves for 18 tissues, goodness of fit is reported in each subplot. The 18 cancers exhibit a good goodness of fit when using *R*^2^ between model-reported age incidence and the empirical cumulative cancer incidence are used as metrics.

We performed extensive simulations and parameter fittings for the empirical incidence cuminc_*c*_(*a*) of cancer type *c* at age *a* using the scaled Weibull function: cuminc_*c*_(*a*) ≈ *P*_*c*_ ⋅ Weibull(*λ*_*c*_, *k*_*c*_)(*a*). The model agrees excellently with age-dependent age incidences of 18 common solid cancers (*R*^2^ > 0.99, Fig. 1).

**Figure 2:**
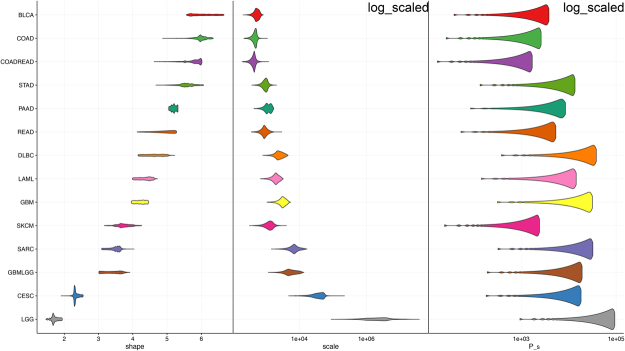
Sensitivity analysis of parameter estimates using the scaled Weibull function for exemplary 14 cancer types. Whereas the estimates *P*_*c*_ for the cell population at risk and the scale parameter *λ*_*c*_ can vary over two order of magnitude, the estimates of the shape parameter *k*_*c*_ are within about ±1. Notice that the shape parameter allows interpretation as the number of limiting events.

Goodness of fit maintains when parameters *P*_*c*_ and *λ*_*c*_, varying roughly two orders of magnitudes (Fig. 2). This finding suggests that many parameter combinations provide similar dynamics that are consistent with empirical data. So any interpretations of *P*_*c*_ and *λ*_*c*_ have to take into account this considerable uncertainty. Nevertheless, the estimates for *P*_*c*_ are several orders of magnitude smaller than the realistic number of stem cells provided by Tomasetti and Vogelstein^8^, yielding evidence supporting the above statement that the number of independent local processes is not equal to the number of stem cells.

**Figure 3:**
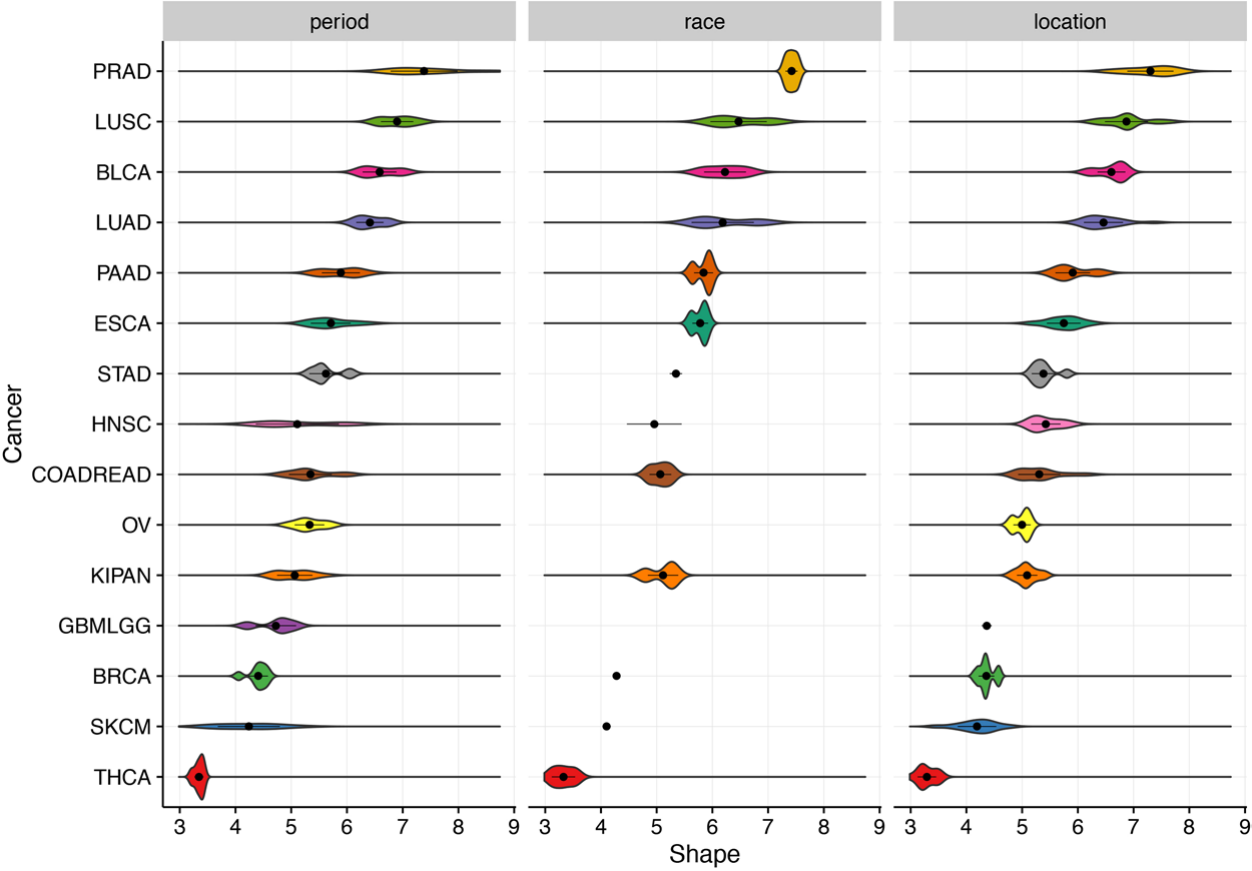
Shape parameters estimated by fitting empirical cancer incidence data using the Weibull function (data with ages for up to 85 years). Cancer patients are grouped by year of diagnosis, race and registry. Cancers are ordered by median values of shape. Shapes are uniform regardless of risk factors, which is consistent with intuitive expectations: race and environmental changes are less likely to alter the number of driver events for cancer onsets.

The estimates of parameter *k*_*c*_, which corresponds to the number of driver events in the mechanistic model, are robust against variations of parameters *P*_*c*_ and *λ*_*c*_ (Fig. 2). Moreover, the estimates of *k*_*c*_ are robust against race, sex, period and location (Fig. 3). In Supplemental Fig. 2 the best fits of shape parameters are plotted against the best fits of scale parameters for 694 time series.

### Relationship between cancer incidence and stem cell divisions

**Figure 4:**
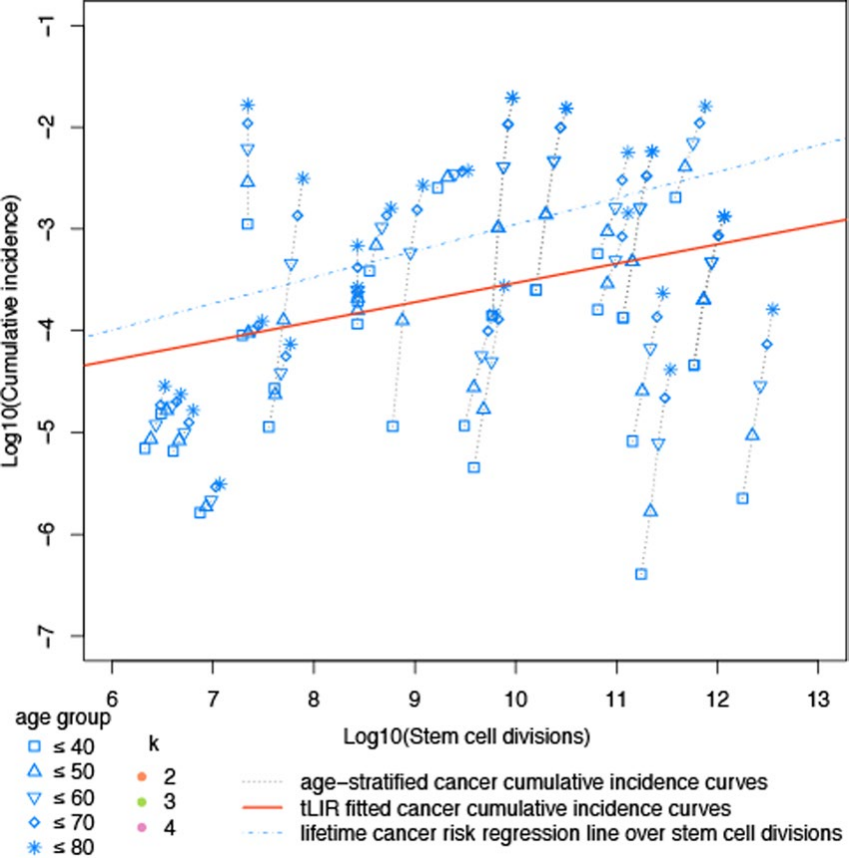
Relationship between cancer incidence and stem cell divisions among 30 cancer types. The lifetime cancer risk regression line is conceptually the same as that used by Wu *et al*.^11^.

Tomasetti and Vogelstein^8^ suggested that the variation in cancer risk among tissues can be explained by the number of stem cell divisions. They reported that the tissue-specific cancer risk is strongly correlated (0.81) with life-time stem cell divisions (LSCD). These authors stated that the total number of stem cell divisions is a *causative factor* of cancer risk. This assumption yields a prediction on age structured data: for tissue type *c* the number of stem cell divisions up to age *a*, which we will denote by LCSD_*c*_(*a*), should then be strongly correlated with cuminc_*c*_(*a*). However, using age incidence data obtained from the SEER-database^27^ we found that the regression lines for most tissue types *c* for age data of 40, 50, 60, 70, and 80 years of cuminc_*c*_(*a*) plotted against LCSD_*c*_(*a*) in a log-log-scale are much steeper than the ones of the regression lines for different *c* and cuminc_*c*_(80)—using 80 as average life span as was done by Tomasetti and Vogelstein^8^ (see Fig. 4).

**Figure 5:**
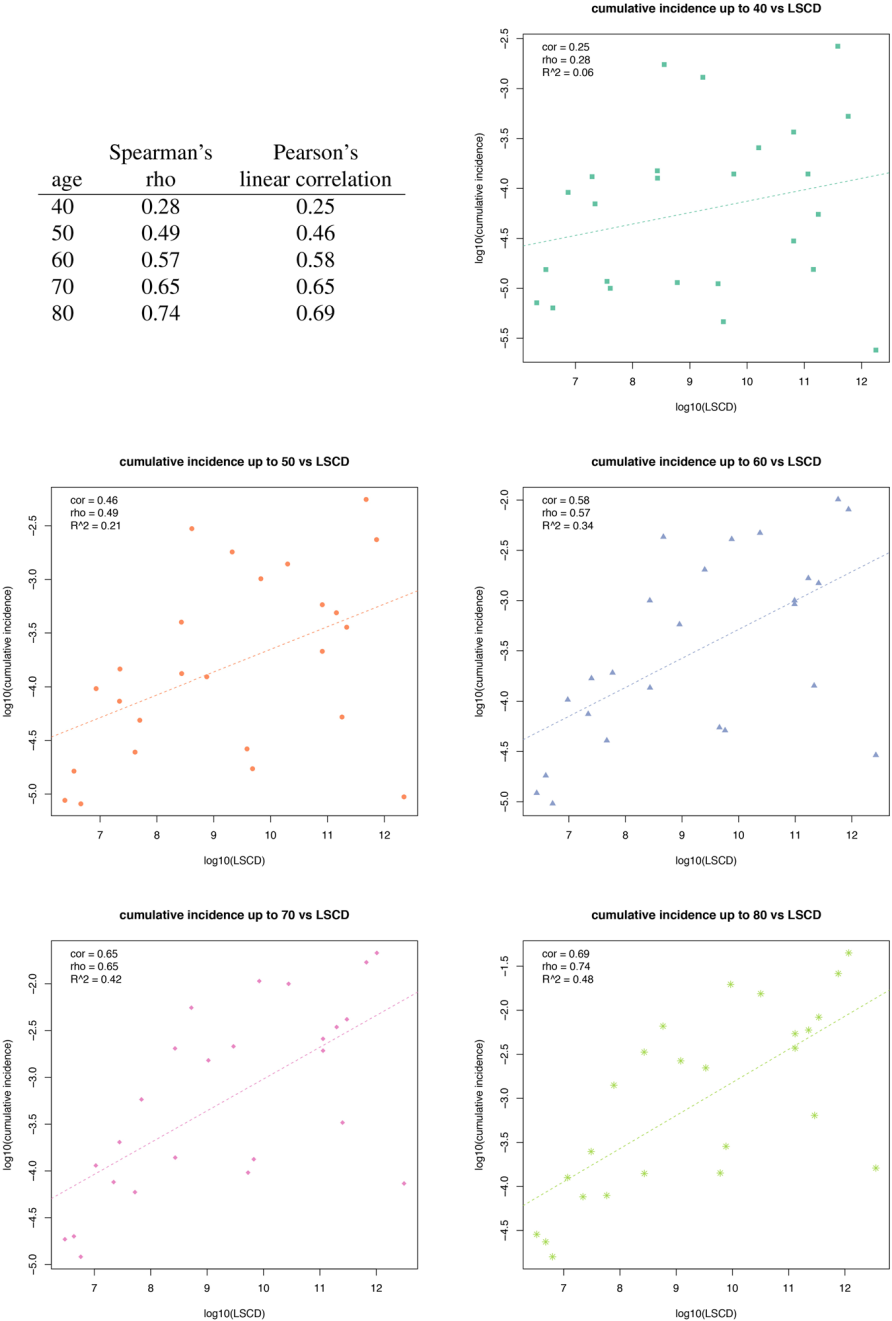
Relationship between cumulative cancer incidences up to age 40, 50, 60, 70, 80 years old and life time stem cell divisions.

This “life time cancer risk” moderately associates with age-dependent stem cell divisions, if one takes a life-time *a* that is less than 70 years (Fig. 5).

Overall, age-dependent stem cell divisions (using ages 40–80 years) is modestly correlated to age-dependent cancer risk for the 31 cancer types considered by Tomasetti and Vogelstein^8^ using the SEER database and the estimates of stem cell divisions given therein (Pearson correlation coefficient *ρ* = 0.51).

Hence, the strong correlation for (life-time) tissue-specific cancer risk with life-time stem cell divisions (LSCD) cannot be explained by the simple causative factor (involving the product of the number of stem cells and the number of divisions of each stem cell) suggested by Tomasetti and Vogelstein^8^. A causal explanation on cancer risk should at least shows that the association between cancer risk and risk factor observed at overall level is reproducible on age stratified data. However, one caveat to such explanation, co-factors of risk factors might not be appreciated.

In our 3-parameter model, which gives good fits for age dependent cancer risks, several relations between the model parameters and cancer risks at a certain age can be observed. For instance in our parameter estimates good fits are possible when taking the inverse of the lifetime cancer risk *P*_*c*_ ≈ 1/cuminc_*c*_(85). However, we will not suggest that the number *P*_*c*_ of cell population at risk is an explanation for the variation of cancer risks among tissues: as the range of *P*_*c*_ yielding good fits varies by two orders of magnitude and independently determining this number is difficult to achieve, a corresponding hypothesis is difficult to verify or to falsify.

As the sensitivity analysis for the scale parameter *λ*_*c*_ (Fig. 2) shows that this parameter varies over several order of magnitudes, still yielding very good fits (*R*^2^ > 0.99), the corresponding estimates for the mutation rate *r* in the tLIR model using the approximation (1) and relation (2) are also very uncertain, even when fixing values of *S* and *d* and leaving out the considerable uncertainty of these. Nevertheless, when using estimates of *S* and *d* taken from the literature^8^ the obtained ranges of values of *r* using relation (2) for several cancer types do not intersect the range [10^−10^, 10^−6^] of “plausible values” of *r* suggested by Wu *et al*.^11^. If we extend the analysis to allow “good fits” by setting a threshold *R*^2^ > 0.85, then good fit of the tLIR model with *r* ∈ [10^−10^, 10^−6^] are possible to achieve (Table 1).

**Table 1:** One possible combination of parameters with which the tLIR model of^11^ fits empirical data well. We are restricting *r* to be in the range [10^−10^, 10^−6^] as was done by^11^.

Cancer	k	r	*R* ^2^	Stem cell	Division rate	Generation^1^	Risk
AML	4.8	1.000e − 06	1.00	1.35e + 08	12.000	1047.01	4.651e − 03
BCC	4.5	1.000e − 06	0.98	5.82e + 09	7.600	678.44	2.181e − 04
CLL	4.9	1.000e − 06	0.99	1.35e + 08	12.000	1047.01	6.925e − 03
COAD	5.5	5.012e − 07	1.00	2.00e + 08	73.000	6232.58	5.677e − 02
DUAD	5.3	1.000e − 06	1.00	4.00e + 06	24.000	2061.93	3.714e − 04
ESCA	4.5	5.012e − 07	0.99	8.64e + 05	17.400	1498.72	3.106e − 03
GBNPAD	3.5	1.000e − 06	0.85	1.60e + 06	0.584	70.25	1.896e − 03
GBM*				1.35e + 08	0.000	27.01	3.825e − 03
HNSC	3.8	1.995e − 07	0.99	1.85e + 07	21.500	1851.64	1.730e − 02
LHCA	3.6	1.000e − 06	0.94	3.01e + 09	0.912	109.05	7.079e − 03
LUAD	2.8	7.943e − 08	0.83	1.22e + 09	0.070	36.13	2.304e − 02
MBM*				1.36e + 08	0.000	27.02	1.414e − 04
SKCM	3.8	1.000e − 06	1.00	3.80e + 09	2.480	242.62	3.038e − 02
OSARC	1.0	1.585e − 08	0.96	4.18e + 06	0.067	27.69	2.696e − 04
OSARCA	1.0	6.310e − 07	0.96	6.50e + 05	0.067	25.01	2.527e − 05
OSARCH	3.0	1.000e − 06	0.99	8.60e + 05	0.067	25.41	1.660e − 05
OSARCL	1.0	3.981e − 07	0.96	1.59e + 06	0.067	26.30	1.312e − 04
OSARCP	3.1	1.000e − 06	0.91	4.50e + 05	0.067	24.47	3.229e − 05
OVGC*				1.10e + 07	0.000	23.39	7.638e − 05
PDAD	3.8	1.000e − 06	0.92	4.18e + 09	1.000	116.96	1.016e − 02
PECA	3.6	1.000e − 06	0.99	7.40e + 07	1.000	111.14	1.498e − 04
SIAD	5.0	5.012e − 07	1.00	1.00e + 08	36.000	3086.58	8.013e − 04
TGCC	1.9	7.943e − 07	0.96	7.20e + 06	5.800	515.78	2.244e − 03
TPFC	3.1	1.000e − 06	0.98	6.50e + 07	0.087	33.35	6.922e − 03
TMCA	3.1	7.943e − 07	0.93	6.50e + 06	0.087	30.03	8.707e − 05

^1^Assuming lifetime is 85 years old, stem cells go through ${\mathrm{log}}_{2}S+d\cdot 85$ generations.

*Cancers of which parameter estimates are impossible because division rate is 0.

### Testing performance of our 3-parameter against other simpler model

**Figure 6:**
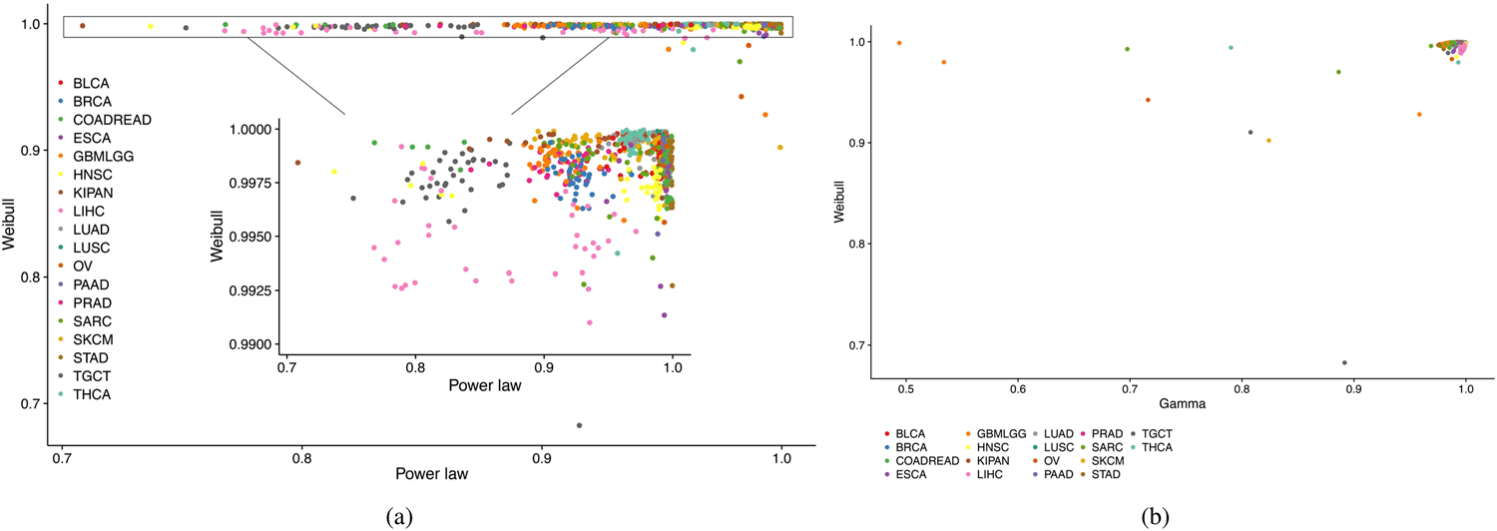
Goodness of fit for scaled Weibull function versus that of power law function (**a**), and scaled Gamma function (**b**). Each dot represents *R*^2^ for one cancer subtype defined by the combination of cancer type and one factor such as diagnosis year, race, location and sex. Cancer types are color coded.

For testing the performance of our 3-parameter model against other simpler models, we compared the fitting performance of the scaled Weibull function to that of 2-parameter power law model arising as the simplest instance from multistage theory^5,7,12,13^. The empirical time series for different locations, periods and races were fitted (694 time series all together) using both models, the power law model had a goodness of fit of *R*^2^ < 0.90 for 90 time series (13.0%), *R*^2^ < 0.95 for 257 time series (37.0%), and *R*^2^ < 0.98 for 366 time series (52.7%). In contrast, our 3-parameter scaled Weibull model resulted in *R*^2^ > 0.9 for all time series, *R*^2^ > 0.98 for 686 (=98.8%) of the time series, and *R*^2^ > 0.99 for 679 (=97.8%) of them (Fig. 6(a)).

We compare the fitting performance of the scaled Weibull function against that of the scaled Gamma function. Although both functions fit data equivalently well in most cases, the scaled Weibull function outperforms the scaled Gamma function in several time series. (Fig. 6(b)) displays *R*^2^ reporting goodness of fit for the two functions. We also calculate the Akaike information ciriterion(AIC)^28^, a likelihood based measurement. A lower AIC value indicates a better fit. Table 2 reports the AIC for 18 cancer types, the AIC values for Weibull function are lower than those of Gamma function in 15 cancer types.

**Table 2:** AIC of the scaled Gamma function and the scaled Weibull function.

Cancer		Gamma	Weibull
LUSC	lung squamous cell carcinoma	2467641.74	2439830.08
LUAD	lung adenocarcinoma	1773103.18	1757914.86
KIPAN	pan − kidney cohort (kich + kirc + kirp)	1086052.13	1074221.46
BLCA	bladder urothelial carcinoma	1397493.34	1364300.97
THCA	thyroid carcinoma	970479.60	966651.56
PAAD	pancreatic adenocarcinoma	684511.50	680353.75
ESCA	esophageal carcinoma	426913.10	424019.74
OV	ovarian serous cystadenocarcinoma	249900.19	248282.96
SKCM	skin cutaneous melanoma	3200014.23	3147559.74
STAD	stomach adenocarcinoma	438574.51	431801.81
PRAD	prostate adenocarcinoma	5411659.76	5425983.72
COADREAD	colorectal adenocarcinoma	3498130.92	3467772.58
GBMLGG	glioma	433655.37	413968.25
BRCA	breast invasive carcinoma	6654036.96	6705665.03
SARC	sarcoma	248310.46	238833.58
TGCT	testicular germ cell tumors	128517.41	128593.83
HNSC	head and neck squamous cell carcinoma	627828.32	626670.00
LIHC	liver hepatocellular carcinoma	510889.67	505268.40

### Estimating the Number of Driver Mutations for Cancer Onset

**Figure 7:**
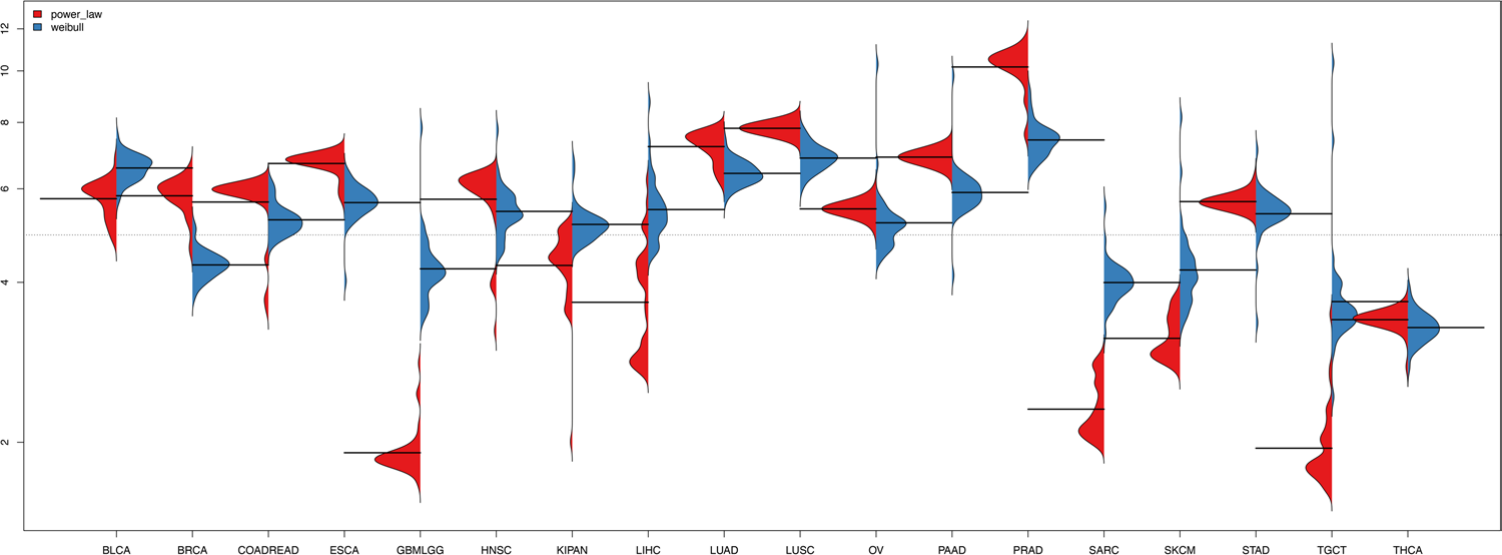
Number of driver mutations required for cancer onset estimated by classical power law model (red) and our scaled Weibull model (blue).

In our model the shape parameter *k*_*c*_ reflects the number of mutations required for cancer onset. The values of this parameter are, however, higher than the number of mutations estimated from sequencing data by Vogelstein *et al*.^29^. Vogelstein *et al*. suggested technical issues as an explanation for the inconsistency between estimates from epidemiological data and sequencing data. Notably, our *k*_*c*_ estimates and the number of driver mutations estimated from a classical power law model are roughly in the same numerical range (Fig. 7). Since we obtain better and more robust fits than the power law model, we believe that our estimated driver mutation numbers are more trustworthy.

## Discussion

In this study we connected the mechanism-based cancer development tLIR model to the Weibull distribution function. We tested its validity by fitting a 3-parameter Weibull function to data from 18 common solid cancer types, consisting of more than 600 time series. The scaled Weibull function fits well with age dependent incidence curves of all studied cancers and outperforms other models, such as the commonly used 2-parameter power law model and a 3-parameter scaled Gamma function model. With the scaled Weibull function, we can estimate the number of driver mutations required for cancer onset in individual cancer types. To our knowledge, this is the first work matching pan-cancer incidence curves with a statistical distribution function that is partially biologically informative.

Compared to the tLIR model developed by Wu *et al*.^11^ we see two technical benefits of our suggested approach: First, the scaled Weibull function involves less parameters than tLIR, but it remains to be biologically interpretable. The tLIR model includes several details of the multi-staged process of cancer development, e.g. the number of steps required for transforming a normal cell to malignancy, the number of stem cells in a tissue and division rate of stem cells. Although the tLIR model indeed provides useful insights into linking age-dependent somatic mutations to cancer risk, it has also limitations. For example, it ignores the effects of clonal expansion^25^. Another issue is that most parameters in the tLIR model are difficult to measure accurately in practice. Following Tomasetti and Vogelstein^8^ the number of stem cell divisions can be estimated, but the accuracy has been criticized^30^. In contrast, our suggested model requires less specific assumptions about the parameters to be measured in practice. Moreover, the Weibull distribution is a special case of the generalized extreme value distribution (GEV) which is well connected to classical statistical approaches to describe rare events^26^.

Our analysis results mostly agree with those provided by Wu *et al*.^11^. They defined intrinsic cancer risk as the probability that one tissue transforms from normal to tumor because of accumulated mutations, and extrinsic cancer risk as 1–intrinsic cancer risk. They quantified upper bounds to intrinsic cancer risk by tLIR but did not properly fit tLIR to epidemiological data, concluding intrinsic factor insignificantly contributes to cancer. According to our understanding their argument mainly results from insufficient exploration of parameter space and implicitly assumes that all tissues require the same number of driver mutation to initiate cancer. Our results suggest that the contribution of extrinsic factors to cancer is overestimated by Wu *et al*.^11^. However, one should note that the excellent agreement between the scaled Weibull distribution function and empirical data does not necessarily exclude that in addition to intrinsic there are further extrinsic and unknown risk factors. In that context it is worthwhile to mention that our estimated number of driver mutations required for cancer onset differs from tissue to tissue. Although the exact number is not validated by biological experiment, this observation is consistent with findings in genetic studies^29^.

One interesting observation is that all non-reproductive tissues have a similar cancer risk accumulation pattern. Cancer incidence rates increase dramatically at about 40–50 years, peaking at about 55–70 years and then decrease. This pattern matches findings reported by Podolskiy *et al*.^31^. A question for future work is whether mutation load agrees with the scaled Weibull function or age-specific mutational signatures^32–34^. Another interesting observation is that testicular germ cell cancer incidence peaks at younger age compared to other cancer types, which might be explained by accelerated aging of testis^31^. Altogether we believe that our suggested approach provides insights into cancer development by providing a link between empirical data and a mechanism-based model.

## Methods

### Fitting cumulative cancer incidence with a model for theoretical intrinsic cancer risk (tLIR)

Wu *et al*.^11^ provided the following recursion formula to compute the chance that a single stem cell acquires *k* mutation hits after *g* divisions given a mutation rate *r*.

3 $$\{\begin{array}{rcl}P({X}_{g+1}=i) & = & \sum _{j=0}^{i}P({X}_{g+1}=i|{X}_{g}=j)P({X}_{g}=j)\\  & = & \sum _{j=0}^{i}(\begin{array}{c}k-j\\ i-j\end{array}){r}^{i-j}{\mathrm{(1}-r)}^{k-i}P({X}_{g}=j)\quad \quad ({\rm{if}}\,i\ne 0\wedge i\ne k)\\ P({X}_{g+1}=\mathrm{0)} & = & {\mathrm{(1}-r)}^{k}P({X}_{g}=\mathrm{0)}\\ P({X}_{g+1}=k) & = & \sum _{j=0}^{k}{r}^{k-j}P({X}_{g}=j)\end{array}$$

given the initial cell state at generation 0:

$$P({X}_{0}=\mathrm{0)}=\mathrm{1;}\,P({X}_{0}=\mathrm{1)}=\mathrm{0;}\ldots ;\,P({X}_{0}=k)=0.$$

Here *X*_*g*_ is accumulated driver mutations at generation *g*, *i* and *j* represents accumulated driver mutations at generation *g* and *g* + 1, respectively. A fully developed tissue with *S* stem cells must go through $n={\mathrm{log}}_{2}S+d\cdot a$ rounds of divisions, assuming division rate is *d* and age *a*. With this transition probability (3), the theoretical lifetime intrinsic cancer risk (tLIR) is formulated as

4 $$\begin{array}{l}{\rm{tLIR}}=1-{\mathrm{(1}-P({X}_{n}=k))}^{S}\end{array}$$

Although the recursion formula being dependent on more than one parameter cannot directly be solved in closed form by standard algorithmic techniques, it has nevertheless a simple closed form solution, which was derived by hand computations and verified by standard symbolic computations (using the computer algebra system Maple 2015.2):

$${\mathrm{(1}-{\mathrm{(1}-r)}^{g})}^{k}$$

The formula for the age-parameterized theoretical lifetime intrinsic cancer risk (tLIR) hence has the following simple closed form solution, which allows much faster and hence more extensive computations and extends the range of admissible values of *k* from the positive integers to the positive real numbers:

5 $$\begin{array}{l}{\rm{tLIR}}(a)=1-{\mathrm{(1}-{\mathrm{(1}-{\mathrm{(1}-r)}^{{\mathrm{log}}_{2}S+d\cdot a})}^{k})}^{S}\mathrm{.}\end{array}$$

Notice that our result basically coincides with the one obtained by Calabrese and Shibata^35^ that was obtained by a direct probabilistic reasoning.

### Relating the tLIR model to a scaled Weibull function

We found a connection between

6 $${\rm{tLIR}}(a)=1-{\mathrm{(1}-{\mathrm{(1}-{\mathrm{(1}-r)}^{{\mathrm{log}}_{2}S+d\cdot a})}^{k})}^{S},$$

and the scaled Weibull function

7 $$1-{\mathrm{(1}-{\rm{Weibull}}(\lambda ,k)(a))}^{P},$$

where *P* is the cell population at risk.

To see this connection, we assume that $r\ll 1$. Then

$${f}_{0}={\mathrm{(1}-{\mathrm{(1}-r)}^{{\mathrm{log}}_{2}S+d\cdot a})}^{k}={\mathrm{(1}-\exp (\mathrm{log}\mathrm{(1}-r)({\mathrm{log}}_{2}S+d\cdot a)))}^{k},$$

and using the Taylor series for log and exp, we obtain

8 $${f}_{0}\approx {(rd)}^{k}{({d}^{-1}{\mathrm{log}}_{2}S+a)}^{k}\mathrm{.}$$

We have

9 $${\rm{tLIR}}(a)=1-{\mathrm{(1}-{f}_{0})}^{S}\mathrm{.}$$

Comparing (7) and (9) we observe that these relations coincide if

$${\mathrm{(1}-{f}_{0})}^{S}={\mathrm{(1}-{\rm{Weibull}}(\lambda ,k)(a))}^{P}={(\exp (-{(\frac{a}{\lambda })}^{k}))}^{P}\mathrm{.}$$

Since for small *f*_0_ > 0 we have 1 − *f*_0_ = exp(−*f*_0_), the last equation can rewritten as

$${f}_{0}^{S}={(\frac{a}{\lambda })}^{kP}$$

Using that relation and (8) one finds

$${(rd)}^{k}{({\mathrm{log}}_{2}S/d+a)}^{kS}\approx {(\frac{a}{\lambda })}^{kP}\mathrm{.}$$

So we have obtained a shifted Weibull distribution. However, if we remove ${d}^{-1}{\mathrm{log}}_{2}S$ from the left hand side of the last equality assuming that

$$d\cdot a\gg {\mathrm{log}}_{2}S$$

we obtain an unshifted one. This condition admits a transparent interpretation, namely, the number of stem cell divisions (for a fixed cell) should be more than the logarithm of stem cell number. Then we have that the tLIR incidence approximately equals to the scaled Weibull incidence if the parameters satisfy

10 $$S{(rd)}^{k}={\lambda }^{-k}P\mathrm{.}$$

Notice that using a Poisson approximation [12, p. 104] we finally obtain

11 $$1-{\mathrm{(1}-{\rm{Weibull}}(\lambda ,k)(a))}^{P}\approx P\,{\rm{Weibull}}(\lambda ,\,k)(a\mathrm{)).}$$

### Stem cell data

Tomasetti and Vogelstein^8^ collected stem cell information for 31 cancer types, including stem cell division rate, stem cell number, tissue total cell number. We excluded 6 from 31 cancer types due to lack of age incidence data: colorectal adenocarcinoma in familial adenomatous polyposis (FAP) patients, colorectal adenocarcinoma in patients with hereditary non-polyposis colorectal cancer (HNPCC, also called lynch syndrome), duodenal adenocarcinoma in FAP patients, head and neck squamous cell carcinoma with human papillomavirus (HPV), hepatocellular carcinoma with hepatitis C virus infection (HCV), lung adenocarcinoma in smokers. Among the 25 remaining cancer types, stem cell information were obtained from supplementary materials of Tomasetti and Vogelstein^8^. We discuss life time stem cell division (LSCD) hypothesis and extrinsic risk factor hypothesis for 25 remained cancers: AML, acute myeloid leukemia; BCC, basal cell carcinoma; CLL, chronic lymphocytic leukemia; COAD, colorectal adenocarcinoma; DUAD, duodenum adenocarcinoma; ESCA, esophageal squamous cell carcinoma; GBNPAD, gallbladder non papillary adenocarcinoma; GBM, glioblastoma; HNSC, head and neck squamous cell carcinoma; LHCA, hepatocellular carcinoma; LUAD, lung adenocarcinoma; MBM, medulloblastoma; SKCM, melanoma; OSARC, osteosarcoma; OSARCA, osteosarcoma of the arms; OSARCH, osteosarcoma of the head; OSARCL, osteosarcoma of the legs; OSARCP, osteosarcoma of the pelvis; OVGC, ovarian germ cell; PDAD, pancreatic ductal adenocarcinoma; PECA, pancreatic endocrine (islet cell) carcinoma; SIAD, small intestine adenocarcinoma; TGCC, testicular germ cell cancer; TPFC, thyroid papillary or follicular carcinoma; TMCA, thyroid medullary carcinoma.

### Cancer incidence data

SEER-9 registries (1973–2013), SEER-4 registries (1992–2013), SEER-5 registries (2000–2013) data were downloaded from Surveillance, Epidemiology, and End Results Program (SEER) database^27^. SEER database covers about 28% USA population, involving more than 100 features such as race, sex, period, location, histology and ICD (international classification of disease) code. These data were stored in ASCII file, we used the SEERaBomb R package to parse them into sqlite file facilitating data manipulation.

Cancer names provided by Tomasetti and Vogelstein^8^ can not be directly mapped into those in SEER database. We addressed this difficulty by two steps: first, annotate tumor primary site to (international classification of disease-oncology 3) ICD-O-3 code based on the literal sense of site in Tomasetti and Vogelstein^8^; second, annotate histology to ICD-O-3 code based on the literal sense of cancer histology by Tomasetti and Vogelstein^8^. For instance, primary site of lung adenocarcinoma is lung, corresponding to ICD-O-3 site code: C340, C341, C342, C343, C348, C349; adenocarcinoma of lung cancer corresponds to ICD-O-3 histology code 8140, 8141, 8143, 8147, 8570, 8571, 8572, 8573, 8574, 8575, 8576. The dictionary needed for mapping step (we call it ICD dictionary) can be found in http://seer.cancer.gov/icd-o-3/. Osteosarcoma definition can be found in ICD dictionary, it is a subtype of malignant bone neoplasm, corresponding ICD-O-3 histology code: 9180–9189. However, the ICD dictionary does not differentiate between osteosarcoma detected in the head, leg, or arm. The ICD9Data database (http://www.icd9data.com/) defines bone cancer using ICD9 code 1700–1709, bone cancer in head, arms, legs, pelvis using ICD9 code 1700, 1704–1705, 1707–1708, 1706 respectively. Head and neck squamous cell carcinoma involves tumors located in many sites, ICD dictionary fails to provide its definition. Liao *et al*.^36^ provided ICD9 site code: 1400–1419, 1430–1499, 1600–1619, we then used ICD-O-3 histology code: 8070–8076, 8078 to select squamous cell carcinoma. More detailed cancer definitions using ICD code can be found in Table 3. Two hematopoietic cancers: acute myeloid leukemia and chronic lymphocytic leukemia, are defined using site recode ICD-O-3/WHO 2008 definition (http://seer.cancer.gov/siterecode/icdo3_dwhoheme/index.html).

**Table 3:** Manually curated cancer definitions.

Cancer	Abbr.	Primary site^1^	Histology^2^
Acute myeloid leukemia	AML		9840, 9861, 9865–9867, 9869, 9871–9874, 9895–9897, 9898, 9910–9911, 9920
Basal cell carcinoma	BCC		8090–8095, 8097–8098
Chronic lymphocytic leukemia	CLL		9823
Colorectal adenocarcinoma	COAD	C180-C189, C199, C209-C212, C218, C260	8140–8141, 8143, 8145, 8147, 8210–8211, 8220–8221, 8570–8576
Duodenum adenocarcinoma	DUAD	ICD9 1520	8140–8141, 8143, 8145, 8147, 8210–8211, 8220–8221, 8570–8576
Esophageal squamous cell carcinoma	ESCA	C150-C155, C158-C159	8070–8076, 8078
Gallbladder non papillary adenocarcinoma	GBNPAD	C239	8000–8005, 8010–8015, 8020–8022, 8041, 8043, 8050–8052, 8070–8076, 8078, 8140–8141, 8143, 8147, 8160–8162, 8255, 8480–8481, 8490, 8500–8501, 8503–8504, 8507–8508 8560, 8562, 8570–8576, 8896, 8900–8902, 8980–8982 9590–9591, 9596, 9650–9655, 9659, 9661–9665, 9667, 9670–9671, 9673, 9675, 9680, 9684, 9687–9688, 9690–9691, 9695, 9698–9699, 9701–9702, 9705, 9712, 9714, 9716, 9719, 9724, 9727–9729, 9731, 9734–9735, 9737–9738, 9740–9741, 9750–9751, 9754–9759, 9811–9818, 9823, 9831, 9837, 9965, 9967, 9971, 9975
Glioblastoma	GBM	C710-C725, C753	9440–9441, 9442, 9444
Head and neck squamous cell carcinoma	HNSC	ICD9 1400–1419, 1430–1499, 1600–1619	8070–8076, 8078
Hepatocellular carcinoma	LHCA	C220-C221	
Lung adenocarcinoma	LUAD	C340-C343, C348-C349	8140–8141, 8143, 8147, 8570–8576
Medulloblastoma	MBM	C710-C725, C753	9470–9474
Melanoma	SKCM	C440-C449	8720–8790
Osteosarcoma	OSARC	ICD9 1700–1709	9180–9189
Osteosarcoma of the arms	OSARCA	ICD9 1704–1705	9180–9189
Osteosarcoma of the head	OSARCH	ICD9 1700	9180–9189
Osteosarcoma of the legs	OSARCL	ICD9 1707–1708	9180–9189
Osteosarcoma of the pelvis	OSARCP	ICD9 1706	9180–9189
Pancreatic ductal adenocarcinoma	PDAD	C250-C259	8140–8141, 8143, 8147, 8210–8211, 8255, 8260–8263, 8310, 8480–8481, 8570–8576
Pancreatic endocrine (islet cell) carcinoma	PECA	C250-C259	8150–8157
Small intestine adenocarcinoma	SIAD	C170-C173, C178-C179	8140–8141, 8143, 8145, 8147, 8255, 8260–8263, 8480–8481, 8570–8576
Thyroid papillary or follicular carcinoma	TPFC	C739	8050, 8260–8263, 8330–8333, 8335, 8337, 8340–8347, 8450
Thyroid medullary carcinoma	TMCA	C739	8510
Ovarian germ cell	OVGC	C569	9060–9065
Testicular germ cell cancer	TGCC	C620-C621, C629	9060–9065

^1^Either ICD-O-3 site code or ICD9 code describing tumor primary site is provided.

2ICD-O-3 histology code.

Although we carefully annotated 25 cancer definitions using ICD code, we can not avoid misclassifications. because annotation needs several data sources of which information confidential levels differ from each other. The Cancer Genome Atlas (TCGA) program^37^ is a flag project of cancer research hosted by National Institutes of Health, it provides comprehensive, high-quality molecular and clinical data. Cancer definitions are well annotated using ICD code in TCGA clinic documents. We therefore assume TCGA cancer definitions are precise and extracted definitions of 18 solid tumors (Table 4). With 18 cancer definitions, we selected patients who were diagnosed with cancer after 2000 from SEER-9 registries, SEER-4 registries, SEER-5 registries data to form SEER-18 registries data. As the highest time resolution of SEER data is 1 year, for each year, we took middle age for fitting models, for example, 0 year-old is modified as 0.5 years-old.

**Table 4:** TCGA cancer definitions for 18 cancer types.

Cancer	Abbreviation	Primary site	Histology
Bladder urothelial carcinoma	BLCA	C670-C676, C679	8010, 8070, 8120, 8130, 8260
Breast invasive carcinoma	BRCA	C502-C505, C508-C509	8010, 8013, 8022, 8050, 8090, 8200–8201, 8211, 8401, 8480, 8500, 8502–8503, 8507, 8510, 8520, 8522–8524, 8541, 8575, 9020
Colorectal adenocarcinoma	COADREAD	C180, C182-C189, C199, C209, C494, C809	8010, 8140, 8211, 8255, 8260, 8263, 8480, 8560, 8574
Esophageal carcinoma	ESCA	C151, C153-C155, C159-C160	8070–8071, 8083, 8140, 8211, 8480
Glioma	GBMLGG	C710-C714, C718-C719	9382, 9400–9401, 9440, 9450–9451
Head and neck squamous cell carcinoma	HNSC	C009, C019, C021-C022, C029-C031, C039-C040, C049-C050, C059-C060, C062, C069, C099, C103, C109, C139, C148, C321, C329, C411	8070–8072, 8074, 8083
Pan-kidney cohort (KICH + KIRC + KIRP)*	KIPAN	C649	8260, 8310, 8312, 8317
Liver hepatocellular carcinoma	LIHC	C220	8170–8171, 8173–8174, 8180, 8310
Lung adenocarcinoma	LUAD	C340-C343, C348-C349	8140, 8230, 8250, 8252–8253, 8255, 8260, 8310, 8480, 8490, 8507, 8550
Lung squamous cell carcinoma	LUSC	C340-C343, C348-C349	8052, 8070–8073, 8083, 8140
Ovarian serous cystadenocarcinoma	OV	C480-C482, C569	8440–8441, 8460
Pancreatic adenocarcinoma	PAAD	C250-C252, C258-C259	8020, 8140, 8246, 8255, 8480, 8500
Prostate adenocarcinoma	PRAD	C619	8140, 8255, 8480, 8490, 8500, 8550
Sarcoma	SARC	C029, C169, C186, C402-C403, C471, C480-C481, C490-C496, C498-C499, C540, C542, C549, C559, C569, C631, C649, C701	8800, 8802, 8805, 8811, 8821–8822, 8830, 8850–8851, 8854, 8858, 8890, 8896, 9040–9041, 9043, 9540
Skin cutaneous melanoma	SKCM	C079, C179, C189, C218, C220, C300, C341, C343, C349, C410, C442-C447, C449, C482, C490-C499, C509, C519, C529, C541, C711, C713, C719-C720, C749, C761-C763, C770, C772-C775, C779	8720–8721, 8730, 8742–8744, 8770–8772
Stomach adenocarcinoma	STAD	C160-C163, C165, C169	8140, 8144–8145, 8211, 8255, 8260, 8480, 8490
Testicular germ cell tumors	TGCT	C629	9061, 9070–9071, 9080–9081, 9085
Thyroid carcinoma	THCA	C739	8050, 8260, 8290, 8330, 8340, 8342, 8344, 8350

*KICH, kidney chromophobe; KIRC, kidney renal clear cell carcinoma; KIRP, kidney renal papillary cell carcinoma.

For robustness analysis of parameter estimates we classified each cancer into subgroups based on location, period and race, data of subgroups were separately fitted to the mathematical models.

### Fitting the models to empirical cancer incidence data

As was done in previous work^38^, empirical cancer incidence *I*(*a*) was calculated by

12 $$\begin{array}{l}I(a)=1-\prod _{i=0}^{a}\mathrm{(1}-{p}_{i}),\end{array}$$

where *p*_*i*_ is frequency of people diagnosed with caner at age *i*.

We performed grid search on an extensive parameter space to fit the tLIR model using ${R}^{2}={(\frac{\sum ({x}_{i}-\bar{x})({y}_{i}-\bar{y})}{\sqrt{\sum {({x}_{i}-\bar{x})}^{2}\sum {({y}_{i}-\bar{y})}^{2}}})}^{2}$ as the metrics for goodness of fit, where *x*_*i*_ and *y*_*i*_ is empirical and model-derived cancer incidence respectively, $\bar{x}$ and $\bar{y}$ respectively denotes mean value of *x* and *y*. Results of fits are given in Table 1 showing that there are biologically reasonable parameter combinations that can yield good fits of the tLIR model for most cancer types.

### Data availability

All data used in this study are publicly available. The sources are detailed in the section on methods.

## Electronic supplementary material

Supplementary Figures and Tables
