# Supplementary material for: A Simple 3-Parameter Model for Cancer Incidences
Source: Sci Rep. 2018 Feb 21;8:3388. doi: 10.1038/s41598-018-21734-x (PMC5821839; doi:10.1038/s41598-018-21734-x)
Supplement: Supplementary file 1 — Supplementary Figures and Tables [file 41598_2018_21734_MOESM1_ESM.pdf]

Supplemental Material: A Simple 3-Parameter Model for Cancer Incidences

Xiaoxiao Zhang[1,2,3], Holger Fröhlich[1,4], Dima Grigoriev[5], Sergey Vakulenko[6,7], Jörg Zimmermann[1], and Andreas Günter Weber[8*]

[1]Bonn-Aachen International Center for Information Technology, Dahlmannstraße 2, 53113 Bonn, Germany
[2]Department of Medicine II, Klinikum Rechts der Isar, Technische Universität München, 81675 München, Germany
[3]German Cancer Consortium (DKTK), German Cancer Research Center (DKFZ), 69120 Heidelberg, Germany
[4]UCB Biosciences GmbH, Alfred-Nobel-Straße 10, 40789 Monheim, Germany
[5]CNRS, Mathématiques, Université de Lille, Villeneuve d'Ascq, 59655, France
[6]Institute for Mechanical Engineering Problems, Russian Academy of Sciences, Saint Petersburg, Russia
[7]Saint Petersburg National Research University of Information Technologies, Mechanics and Optics, Saint Petersburg, Russia
[8]Institut für Informatik II, Universität Bonn, Friedrich-Ebert-Allee 144, Bonn, Germany
[*]weber@cs.uni-bonn.de

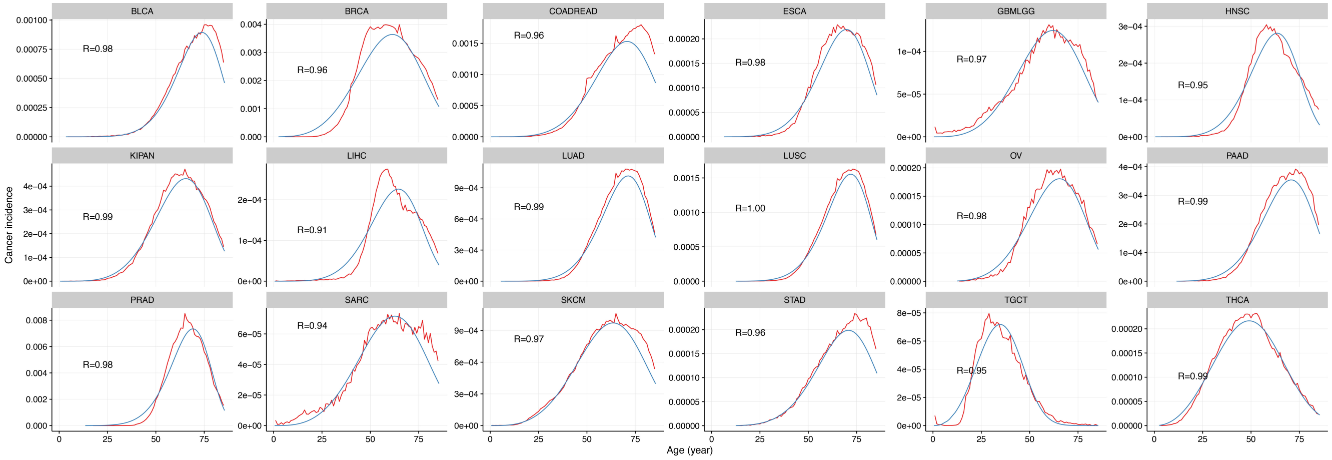

Supplemental Figure 1. Empirical cancer incidence data agree with Weibull probability function well in 18 cancers (data with ages up to 85 years old). Empirical (blue line) and Weibull function fitted (red line) cancer incidence curves for 18 tissues, goodness of fit is reported in each subplot. The 18 cancers have good goodness of fit when using R^2 between model reported age incidence and empirical incidence as metric.

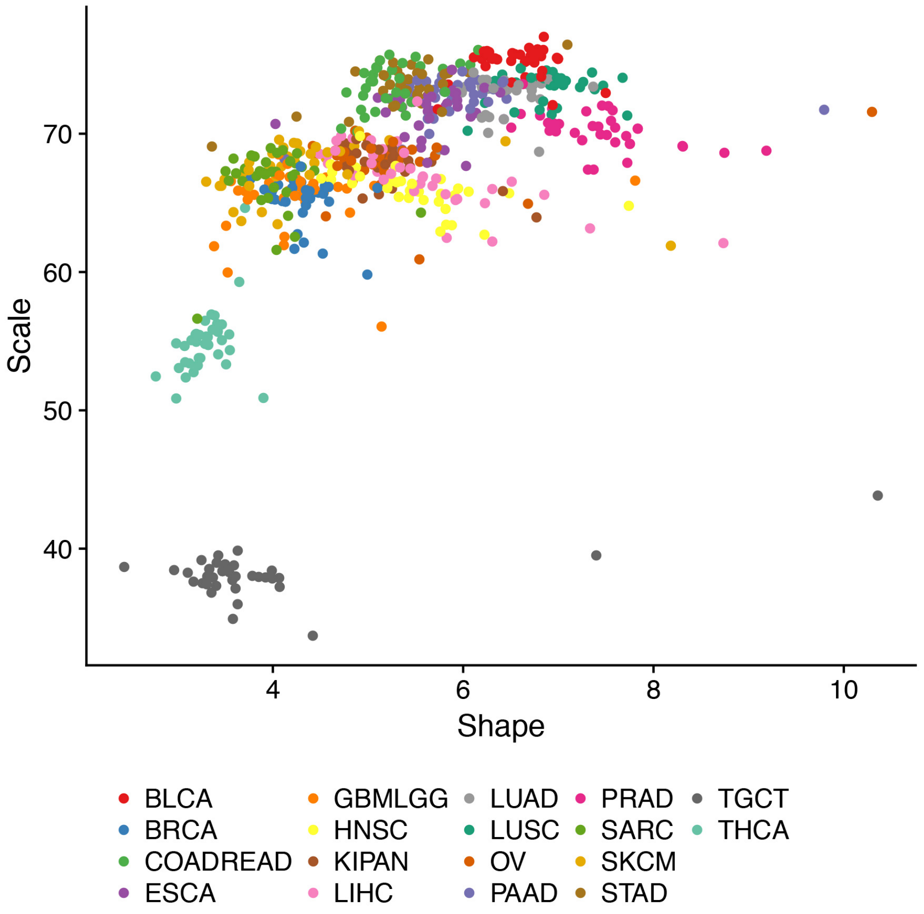

Supplemental Figure 2. Relationship between shape and scale parameters in Weibull function for 694 time series. Each time series corresponds to one cancer subtype defined by the combination of cancer type and one factor like diagnosis year, race, region and sex. Cancer types are color coded. Each dot represents a time series.

Supplemental Table 1. One possible combination of parameters with which tLIR model fits empirical data well *without* restricting r to be in the range $[10^{-10}, 10^{-6}]$ as was done by Wu et al.

Cancer	k	r	R^2	Stem cell	Division rate	Generation[1]	Risk
AML	4.9	1.995e-06	1.00	1.35e+08	12.000	1047.01	4.651e-03
BCC	6.9	5.012e-05	1.00	5.82e+09	7.600	678.44	2.181e-04
CLL	8.0	1.000e-04	1.00	1.35e+08	12.000	1047.01	6.925e-03
COAD	6.9	1.000e-05	1.00	2.00e+08	73.000	6232.58	5.677e-02
DUAD	7.3	6.310e-05	1.00	4.00e+06	24.000	2061.93	3.714e-04
ESCA	8.2	1.585e-04	1.00	8.64e+05	17.400	1498.72	3.106e-03
GBNPAD	9.0	5.012e-04	1.00	1.60e+06	0.584	70.25	1.896e-03
GBM*				1.35e+08	0.000	27.01	3.825e-03
HNSC	7.1	6.310e-05	1.00	1.85e+07	21.500	1851.64	1.730e-02
LHCA	8.7	7.943e-04	1.00	3.01e+09	0.912	109.05	7.079e-03
LUAD	9.0	7.943e-04	0.87	1.22e+09	0.070	36.13	2.304e-02
MBM*				1.36e+08	0.000	27.02	1.414e-04
SKCM	4.3	1.995e-05	1.00	3.80e+09	2.480	242.62	3.038e-02
OSARC	1.0	1.585e-08	0.96	4.18e+06	0.067	27.69	2.696e-04
OSARCA	1.0	6.310e-07	0.96	6.50e+05	0.067	25.01	2.527e-05
OSARCH	4.5	3.162e-05	0.99	8.60e+05	0.067	25.41	1.660e-05
OSARCL	1.0	3.981e-07	0.96	1.59e+06	0.067	26.30	1.312e-04
OSARCP	9.0	1.259e-03	0.97	4.50e+05	0.067	24.47	3.229e-05
OVGC*				1.10e+07	0.000	23.39	7.638e-05
PDAD	8.6	6.310e-04	1.00	4.18e+09	1.000	116.96	1.016e-02
PECA	7.0	7.943e-04	1.00	7.40e+07	1.000	111.14	1.498e-04
SIAD	5.8	1.259e-05	1.00	1.00e+08	36.000	3086.58	8.013e-04
TGCC	4.3	1.000e-04	0.99	7.20e+06	5.800	515.78	2.244e-03
TPFC	8.8	3.981e-03	0.99	6.50e+07	0.087	33.35	6.922e-03
TMCA	8.9	1.000e-03	0.99	6.50e+06	0.087	30.03	8.707e-05

1 Assuming lifetime is 85 years old, stem cells go through $\log_2 S + d \cdot 85$ generations.

* Cancers of which parameter estimates are impossible because division rate is 0.
